# Clinical Implication of Serum Adiponectin Levels in Adult Patients with Atopic Dermatitis

**DOI:** 10.3390/jcm11216255

**Published:** 2022-10-24

**Authors:** Sul-Hee Lee, Youin Bae, Young-Lip Park

**Affiliations:** 1 Department of Dermatology, Soon Chun Hyang University Bucheon Hospital, Bucheon 14584, Korea; 2 Department of Dermatology, College of Medicine, Hallym University, Hallym University Dongtan Sacred Heart Hospital, Hwaseong 18450, Korea

**Keywords:** adiponectin, atopic dermatitis, biomarker

## Abstract

Atopic dermatitis (AD) is characterized by chronic, relapsing, pruritic inflammatory skin disease. Adiponectin has been reported to have anti-inflammatory effects not only on metabolic disorders but also on various inflammatory disorders. The study aimed to validate adiponectin as a potential biomarker for AD disease severity and treatment response. Seventy-five patients with AD and 28 healthy volunteers were enrolled in the study. Patient information, including Eczema Area and Severity Index (EASI) scores and pruritus numeric rating scales (NRSs), were collected. An enzyme linked immunosorbent assay (ELISA) was conducted to measure levels of serum adiponectin. Additionally, sera of patients treated with dupilumab were collected and measured at 16 and 52 weeks from baseline. Serum adiponectin levels were significantly lower in moderate and severe AD patients than in the control and mild AD patients. Serum adiponectin level was negatively correlated with the EASI score and pruritus NRS. However, no significant changes were observed according to biologic treatment for AD. Low serum adiponectin levels are associated with moderate to severe AD, suggesting a potential role for adiponectin as a biomarker for severity assessment of AD.

## 1. Introduction

Atopic dermatitis (AD) is a chronic inflammatory skin disease with a heterogeneous pathogenesis and diverse clinical manifestations. The disease is associated with pruritus in all affected patients and both allergic and non-allergic comorbidities [1]. AD is a disease that requires active intervention since it is often accompanied by sleep disturbance, depression, and anxiety in addition to skin lesions [2]. As a result, AD can have a profound effect on quality of life and lead to a huge socioeconomic burden [3]. Though the etiology of AD is complex, recent advances in understanding genetic predispositions, skin barrier dysregulation, innate and adaptive immune imbalance, and altered skin microbiomes have led to the development of various therapeutic alternatives including molecular-targeted treatments such as biologics and small-molecule inhibitors [4,5,6]. Among them, dupilumab is the first monoclonal antibody against moderate-to-severe AD approved globally. It showed a great efficacy in improving the skin lesions of AD, subjective symptoms, health-related quality of life, and symptoms of anxiety and depression in AD patients [2,7,8].

Meanwhile, efforts are being made to identify reliable biomarkers that can act not only as diagnostic tools, but also reflect the severity of the disease and response to treatment. To date, clinicians have largely relied on clinical tools such as the Eczema Area and Severity Index (EASI), Investigator Global Assessment (IGA), and SCORing Atopic Dermatitis (SCORAD) to assess the severity of AD and treatment responsiveness [9].

A significant number of biomarkers have been discovered and clinically utilized in the assessment of AD severity or treatment outcomes. However, the efficacy of biomarkers in reflecting the severity of the disease varies drastically. Moreover, there has been an ongoing discussion about the association between AD and markers that reflect the presence or absence of systemic diseases [10,11]. In particular, there have been a significant number of studies on the role of adipokine, a cytokine secreted by adipose tissue, in AD. Adipokines are known as primary mediators for adipose-tissue-regulated systemic inflammatory diseases. Interestingly, a recent finding revealed that serum levels of adipokines correlate with the onset and severity of various dermatologic disorders including AD, psoriasis, rosacea, and malignant melanoma [12,13].

In the present study, we aimed to measure serum levels of adiponectin, an anti-inflammatory adipokine, in AD patients with varying clinical severities and compare these with those of a body mass index (BMI)-matched control group. We also aimed to evaluate changes in adiponectin levels in response to dupilumab treatment in patients with severe AD.

## 2. Materials and Methods

### 2.1. Enrollment of Study Subjects

Seventy-five patients with clinically diagnosed AD and 28 healthy volunteers were enrolled in the case control study. AD was diagnosed according to the modified Hanifin and Rajka [14] criteria issued by the Korean Atopic Dermatitis Association.

Inclusion criteria for AD patients were as follows: (1) patients diagnosed with AD for at least 2 years, (2) patients older than 18 years, (3) patients who had not received biologic treatments within the last 1 year, and (4) patients who did not have other dermatologic or systemic disorders except for atopy or allergy related disorders. Twenty-eight healthy volunteers without histories of any allergic disorder or other dermatologic disorder were also included. This study was approved by the Institutional Review Board (IRB) of Hallym University Dongtan Sacred Heart Hospital (IRB file No. HDT 2020-06-025). All participants provided written informed consent.

### 2.2. Clinical and Laboratory Data Collection

Subjects’ clinical characteristics including age, sex, AD duration, and accompanying diseases were collected. BMI was calculated for each subject according to body weight and height (kg/m^2^). Severity scores assessed by the EASI, IGA, pruritus numerical rating scale (NRS), and Dermatology Life Quality Index (DLQI) were obtained from all AD patients and during treatment with dupilumab in the severe AD group. Laboratory data including CBC, routine chemistry, serum total IgE (IU/mL), eosinophil count (#/mm^3^) and lipid profiles were also measured.

Participants were divided into three groups based on BMI (normal weight: <23; overweight: ≥23 and <25; and obese: ≥25) according to the classification of obesity by the Korean Society for the Study of Obesity (KSSO) [15]. AD patients were divided into three subgroups based on EASI scores (mild: <16; moderate: ≥16 and <23; and severe: ≥23), DLQI score (0–5: no or small effect on patient’s life; 6–10: moderate effect on patient’s life; and 11–30: large-to-extremely large effect on patient’s life), and the pruritus NRS according to consensus Korean diagnostic guidelines to define severity classification of AD [16].

### 2.3. Treatment of Severe AD Patients

Among AD patients enrolled, participants classified as having severe AD were treated with dupilumab for more than a year. Their clinical and laboratory data were collected at baseline, week 16, and week 52.

### 2.4. Measurement of Serum Adiponectin Levels

Fasting blood samples were collected from patients and healthy subjects. In addition to conventional blood sampling, 3-mL samples of blood were collected in 5-mL BD vacutainer serum separation tubes for enzyme-linked immunosorbent assays (ELISAs). Serum levels of adiponectin were measured using an ELISA kit (R&D Systems, Minneapolis, MN, USA), according to the manufacturer’s instructions. The resultant color reaction was read at 450 nm using a microplate reader.

### 2.5. Statistical Analysis

Data are expressed as mean ± SDs. Statistical analyses were performed using SPSS Statistics 21 (IBM SPSS Inc., Chicago, IL, USA). Normality was satisfied in each group of AD patients and volunteers. Statistical analyses were conducted with Student’s *t*-tests and one-way ANOVA followed by multiple comparisons with Tukey’s honest significant difference test. Correlations were calculated by Pearson’s correlation analysis. Changes in clinical and laboratory values in the severe AD group before and after treatment were calculated using repeated-measures ANOVA. *p* value of less than 0.05 was considered statistically significant.

## 3. Results

### 3.1. Characteristics of Study Subjects

The general characteristics of the study subjects are presented in Table 1. Of a total of 103 native Korean subjects (56 males and 47 females; mean age, 26.63 ± 6.28 years), the proportions of sexes, ages, BMIs, and lipid profiles (total cholesterol and LDL-cholesterol) were similar between the control and AD groups.

AD patients were divided into mild, moderate, and severe groups. There were significant differences in total IgE levels, EASI scores, IGA scales, and pruritus NRSs between groups. However, blood eosinophil counts showed no significant differences.

### 3.2. Serum Adiponectin Levels Were Lower in the AD and High BMI Groups

**Figure 1:**
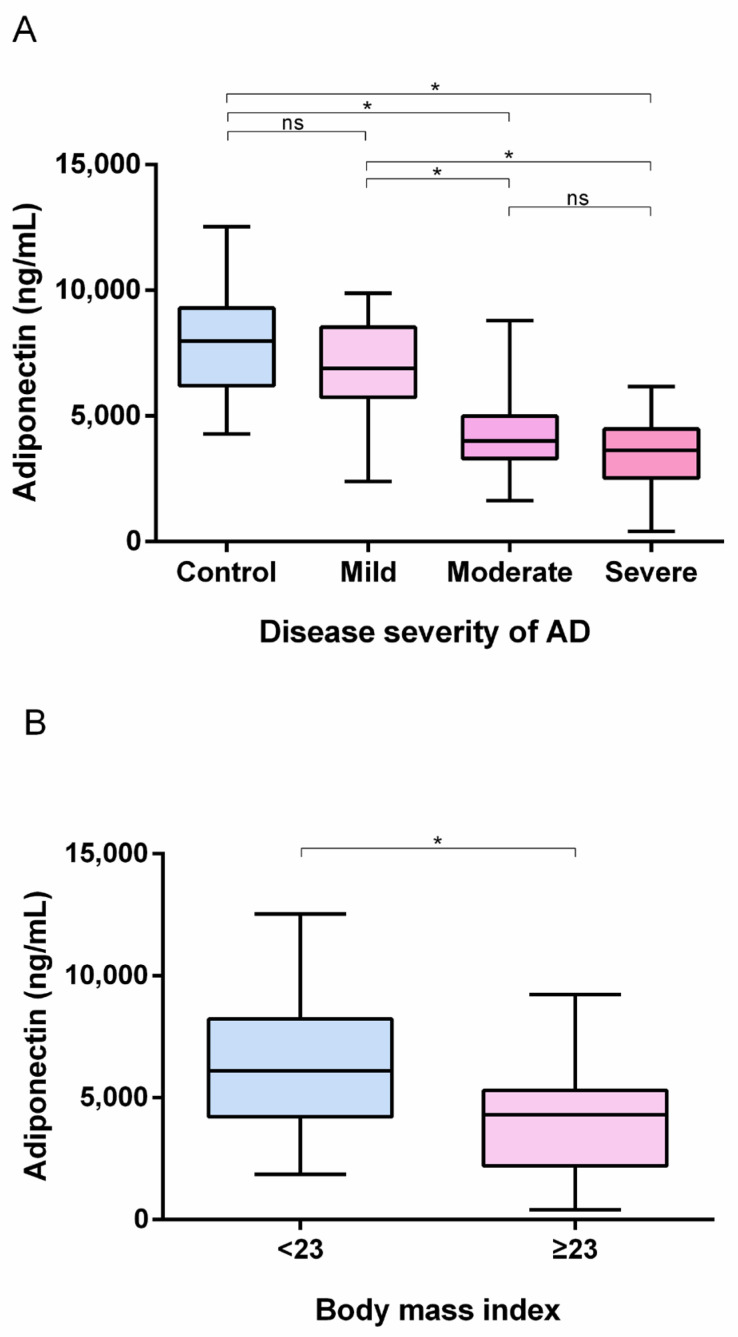
Comparison of serum adiponectin levels between (**A**) each atopic dermatitis group classified by severity and the control group, (**B**) the normal weight group (BMI < 23) and the overweight or obese group (BMI ≥ 23). AD: atopic dermatitis, BMI: Body mass index. * *p* < 0.01.

Adiponectin levels were significantly lower in the AD group compared to the control group (4911 ± 2218 ng/mL vs. 7889 ± 1929 ng/mL, *p* < 0.001). In the subgroup analysis, there were significant differences between the mild (6997 ± 1798 ng/mL) and moderate (4305 ± 1760 ng/mL) groups (*p* < 0.001), and between the mild and severe (3524 ± 1370 ng/mL) groups (*p* < 0.001). However, there were no significant differences between the moderate and severe groups (*p* = 0.08) (Figure 1A).

Also, adiponectin levels were significantly lower in the normal weight group compared to the overweight or obese group (6249 ± 2338 ng/mL vs. 4063 ± 2278 ng/mL, *p* < 0.001) (Figure 1B).

### 3.3. Association of Serum Adiponectin Levels with Various AD Patient Data

**Figure 2:**
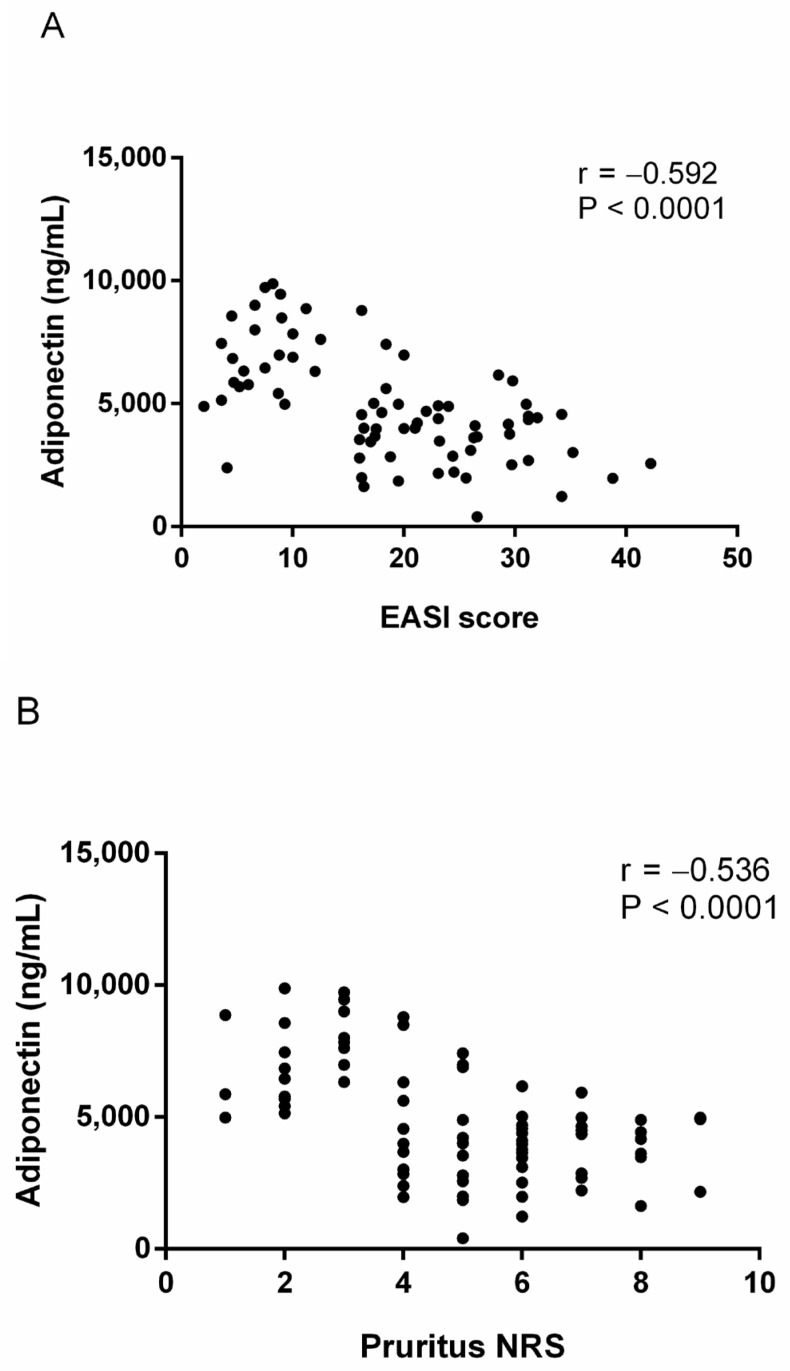

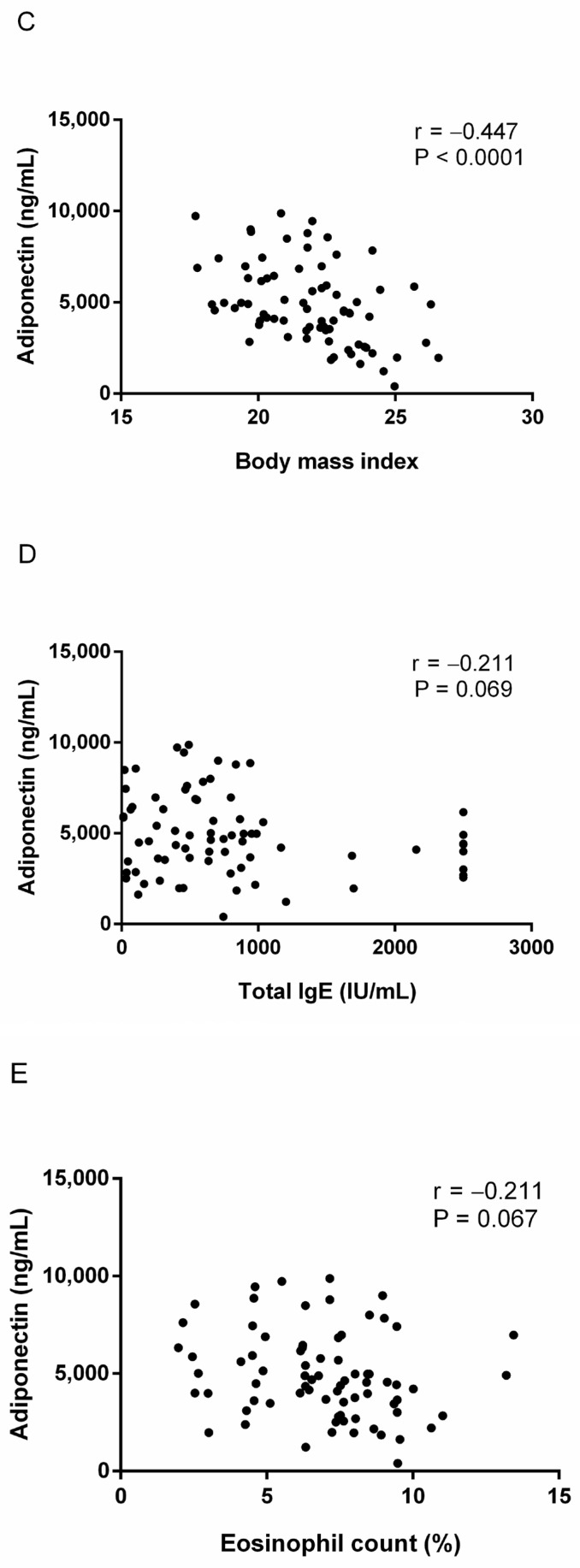
Correlation between serum adiponectin level and various clinical and laboratory values (**A**) EASI score (**B**) pruritus NRS (**C**) body mass index (**D**) total IgE level, and (**E**) blood eosinophil count (%). EASI: Eczema Area and Severity Index, NRS: numeric rating scale.

The relationships between clinical and laboratory values and adiponectin levels in AD patients were analyzed using Pearson’s correlation coefficient. Adiponectin levels were moderately negatively correlated with EASI scores (*r* = −0.592, *p* < 0.001) and pruritus NRS (*r* = −0.536, *p* < 0.001). Also, adiponectin levels showed fair to moderate negative correlations with BMIs (*r* = −0.447, *p* < 0.001). However, adiponectin levels were not correlated with total IgEs and eosinophil counts (Figure 2).

We also analyzed the relationship between clinical score and laboratory values. Correlations between values of BMI, EASI, IGA, pruritus NRS, total IgE, and eosinophil count are presented in Table 2. EASI, IGA and pruritus NRS values were strongly correlated with one another, and clinical severity scores were fairly correlated with total IgEs or eosinophil counts, however, BMIs were not significantly correlated with other clinical and laboratory values.

### 3.4. Changes in Serum Adiponectin Levels According to AD Treatment

**Figure 3:**
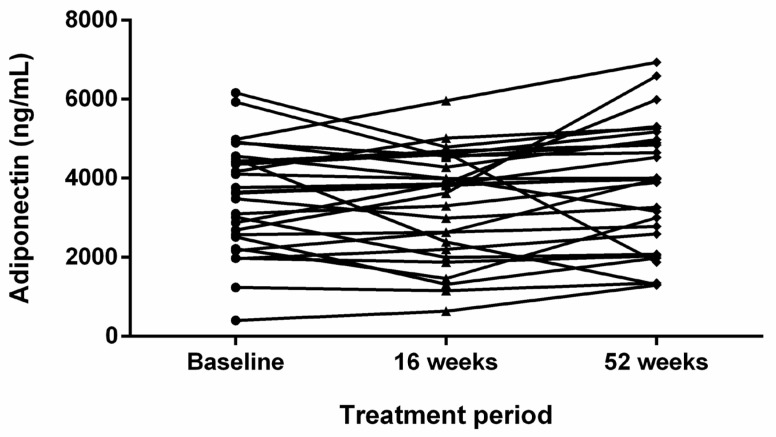
Changes in serum adiponectin levels in response to dupilumab treatment in the group of patients with severe atopic dermatitis.

In the severe AD group, repeated measurements of serum adiponectin level in response to dupilumab treatment (baseline, week 16, and week 52) indicated no significant changes (*p* = 0.18) while the EASI score decreased significantly using repeated-measures ANOVA. (Figure 3) Changes in clinical and laboratory value in response to dupilumab treatment in patients with severe AD are summarized in Table 3.

## 4. Discussion

In this study, we determined whether serum adiponectin levels differ in BMI-matched normal controls and AD patients according to the AD severity. In addition, a prospective study was conducted to determine whether changes in adiponectin levels were observed in the severe AD group that showed clear clinical improvement after treatment with dupilumab, a novel interleukin (IL)-4 receptor alpha antagonist targeting and blocking IL-4 and IL-13 molecules [8].

AD is one of the most common chronic inflammatory diseases of the skin. Its clinical features vary depending on age, race, genetic background, and environmental factors. Therefore, the epidemiology of atopy is inevitably complicated, and it is challenging to identify the etiology and therapeutic approaches accordingly. Nevertheless, recent genetic and proteomic studies have provided a significant level of access to the disease. Importantly, this enhanced understanding of the disease facilitates’ precise target delineation compared with conventional ‘one-size-fits-all’ management paradigms [17].

The most important immunological foundation involved in the onset and exacerbation of AD is the T helper (Th) 2 response; in addition, Th1, Th17, and Th22 response make a widening of adaptive immunity [18]. As AD progresses, various cytokines, such as interleukins, tumor necrosis factor-alpha (TNF-α), and chemokines, released through T cells induce inflammatory reactions, further affecting keratinocytes differentiation [19]. Dysfunction of the skin barrier also plays a major role in the induction and amplification of AD. In the epidermis, keratinocytes in the disrupted barrier can produce alarmins such as thymic stromal lymphopoietin (TSLP), IL-25, and IL-33.

During efforts to find biomarkers related to the exacerbation or improvement of AD, the roles of a series of adipokines in inflammatory skin diseases have been highlighted and studied [20,21,22,23,24,25]. Since adipose tissue functions as an important endocrine organ capable of secreting adipokines including leptin, adiponectin, resistin, and so on, these proteins produced from adipocytes are hallmarks of inflammation [26]. There is accumulating evidence that obesity causes adipose tissue dysfunction, which may induce dysregulation of pro- and anti-inflammatory adipokines, resulting in various systemic metabolic diseases [27,28]. Recent findings suggest that adipokines are also related to various skin diseases such as psoriasis, AD, acne vulgaris, rosacea, and malignant melanoma [13].

Among the various adipokines discovered to date, adiponectin plays a role in the anti-inflammatory response by reducing the production of various inflammatory cytokines in inflammatory skin disease. It reduces the production of TNF-α, IL-6, and interferon-gamma through AdipoR1 and R2 signaling mechanisms [29]. Though the exact role of adiponectin in AD pathogenesis is not fully understood, a recent study reported that adiponectin attenuates inflammation in atopic dermatitis-like reconstructed human epidermis [30]. In this study, the authors found that expression levels of inflammatory mediators such as TSLP, IL-8, and TNF-α were reduced by adiponectin treatment.

The association between adiponectin and various skin diseases has been elucidated in a number of clinical studies. Psoriasis is one of the most studied skin diseases; in one study, adiponectin was inversely correlated with psoriasis severity and significantly increased after treatment [20]. However, this finding was not confirmed in another study [22]. There are also AD studies with contradictory results regarding disease severity and adiponectin levels [25,31]. In the present study, we measured serum adiponectin levels in AD patients and BMI-matched controls. AD patients were grouped by clinical severity assessment tools, including the EASI, DLQI, and pruritus NRS. We found that moderate to severe AD patients had significantly lower adiponectin levels compared with not only controls, but also patients with mild AD. Through these results, we confirmed that adiponectin was reduced in AD patients independently of BMI. Moreover, we could hypothesize that the extent of cutaneous inflammation in AD is a key factor determining serum adiponectin levels.

Additionally, we analyzed the association of adiponectin levels with several clinical and laboratory values. Interestingly, serum adiponectin levels showed moderate negative correlations with EASI scores and pruritus NRSs along with BMI. This result may consolidate the hypothesis that the extent of inflammation is strongly related to adiponectin levels in AD patients.

The second subject of our study was whether adiponectin levels would change in response to AD treatment. Unlike studies of other systemic metabolic diseases or psoriasis, few studies on this topic have been conducted in AD patients. We conducted a prospective study including an observational experiment. Each patient with severe AD received treatment with dupilumab, a monoclonal antibody that blocks IL-4/IL-13 signaling, thereby inhibiting receptor signaling downstream in T cells; these patients exhibited huge decreases in EASI scores. Meanwhile, analysis using repeated measures ANOVA, similar to another study on other inflammatory diseases treated with a TNF-α blockade [32], revealed no changes in adiponectin levels.

In contrast to the reports on psoriasis [20,33], no significant changes in adiponectin levels were observed with improvement of AD after treatment. It seems necessary to focus on the differences in results between studies on psoriasis and AD. The number of studies demonstrated that adiponectin induces anti-Th1 inflammatory effects [34,35,36]. Those anti-inflammatory effects may exert through inhibition of Th1 cytokines such as IFN-γ or TNF-α in AD. We hypothesize that a gradual decrease in Th1 cytokines in response to improvement of AD may lead to an increase in adiponectin by negative feedback.

Although Th1 cells and their derived cytokines are known to play important roles in the chronic phase of AD, however, since Th1 cytokines are not the therapeutic target of dupilumab which inhibits core Th2 cytokines in AD, it can be concluded that it was difficult to expect and an rapid reduction in Th1 cytokines as much as the reduction in clinical scores.

In addition to interpretation of these results, we intend to further elucidate the interaction between adiponectin and TNF-α and establish hypotheses. As adiponectin inhibits the production of TNF-α, which has a potential pro-inflammatory role in both psoriasis and AD, low levels of adiponectin may be expected in both chronic inflammatory skin diseases. However, it should be noted that TNF-α blockades are relatively less effective in AD compared to psoriasis, which has been established as a therapeutic choice along with IL-17 inhibitors and IL-23 inhibitors. Therefore, based on the result that changes in adiponectin levels were not observed after AD treatment, we can hypothesize that TNF-α plays a limited role in the improvement of AD compared with psoriasis.

According to the U.S. Food and Drug Administration (USFDA), a biomarker is “a defined characteristic that is measured as an indicator of normal biologic processes, pathologic processes, or responses to an exposure or intervention, including therapeutic interventions”. To elaborate on this in more detail, it is explained as “molecular, histologic, radiographic, or physiologic characteristics are types of biomarkers.” It can be subdivided by purpose such as diagnosis, monitoring/determination of severity, prognosis/prediction, pharmacodynamics, responsiveness, and safety. (FDA-NIH: Biomarker-Working-Group, 2016).

To date, unlike other chronic diseases, clinicians have largely relied on clinical tools such as Eczema Area and Severity Index (EASI), Investigator’s Global Assessment (IGA), and SCORing Atopic Dermatitis (SCORAD) to assess the severity of AD and treatment response. In this context, efforts are needed to identify serum biomarkers correlating with AD severity and treatment response.

From a broad perspective, biomarkers reflecting disease severity or treatment response in AD may be of the following types. First, based on the immune pathways involved in AD, various Th2-related cytokine and chemokine biomarkers have been discovered. Also, barrier-related proteins, including filaggrin, loricrin, and natural moisturizing factors, are the potential biomarkers inversely related to clinical severity [9]. Markers related to general inflammation or allergies such as C-reactive protein, serum lactate dehydrogenase, periostin, and peripheral eosinophil counts may be included as biomarkers correlated with clinical severity of AD [37,38,39,40]. Adipokines have recently received attention as biomarkers of systemic inflammation and their roles in existing metabolic diseases. In particular, adiponectin is inversely correlated with the EASI and pruritus NRS, tools for evaluating AD clinical severity; this shows that to some extent adiponectin has potential as a biomarker of AD.

Jin et al. [41] reported that adiponectin upregulated filaggrin expression in normal human keratinocytes. Also, Seo et al. [30] highlighted the possible therapeutic application of adiponectin based on their finding that adiponectin increased the mRNA expression of major differentiation proteins and epidermal lipid biosynthetic enzymes in the AD-like epidermis.

In our study, we clinically evaluated adiponectin as a possible biomarker of disease severity and treatment response in AD. Nevertheless, this study has some limitations. First, since we failed to see a decrease in serum adiponectin level in successfully treated patients, additional long-term prospective case control studies should be conducted. Second, to establish adiponectin as a novel biomarker, another validation study based on immunohistochemical analysis of patients’ lesional and non-lesional skin is needed. Third, we did not test a large number of patients and controls for validation of adiponectin. We are planning follow-up studies to evaluate the potential of this protein as a novel biomarker by recruiting a larger number of subjects and using more specific study design and subject subgroupings; for example, with or without obesity or intrinsic or extrinsic AD. Comparative studies with known biomarkers, such as cytokines, chemokines, and differential proteins of epidermis to establish their relationship with adiponectin are also planned.

## 5. Conclusions

In conclusion, in this study, we present adiponectin as a potential clinical biomarker of AD based on a case control study. By measuring serum adiponectin levels, adiponectin was validated as a diagnostic biomarker of moderate to severe AD. Additionally, the relationship between serum adiponectin levels and clinical severity was established with statistical significance within the patient group.

## Figures and Tables

**Table 1:** Clinical characteristics of study subjects.

Characteristic	Control (*n* = 28)	Atopic Dermatitis			*p* Value *
		Mild (*n* = 25)	Moderate (*n* = 22)	Severe (*n* = 28)	
Age	28.93 ± 5.40	27.77 ± 6.45	24.45 ± 7.23	25.66 ± 6.58	NS
Male	15 (53.5)	14 (56.0)	12 (54.5)	15 (53.5)	NS
Disease duration (year)	-	11.39 ± 4.51	15.08 ± 5.87	14.85 ± 6.42	NS
Allergic disorders					
Rhinitis	0	16 (64)	15 (68.2)	20 (71.4)	<0.001
Conjunctivitis	0	6 (24)	8 (36.4)	17 (60.7)	<0.001
Asthma	0	1 (4)	3 (13.6)	6 (21.4)	<0.001
Total IgE (IU/mL)	69.2 ± 124.4	420.7 ± 279.8	825.5 ± 624.1	1140.1 ± 950.6	<0.001
Blood eosinophils count (%)	2.45 ± 1.79	5.81 ± 2.09	7.55 ± 2.72	7.40 ± 2.21	NS
Total cholesterol (mg/dL)	173.5 ± 37.75	169.45 ± 29.87	179.26 ± 36.74	183.74 ± 39.88	NS
LDL-cholesterol (mg/dL)	121.53 ± 25.64	122.02 ± 21.55	130.57 ± 28.63	133.24 ± 30.26	NS
Body mass index	21.53 ± 1.80	21.68 ± 2.12	21.82 ± 1.91	22.19 ± 2.13	NS
<23	24 (85.7)	20 (80)	17 (77.3)	16 (57.1)	
≥23, <25	3 (10.7)	4 (16)	4 (18.2)	10 (35.7)	
≥25	1 (3.5)	1 (4)	1 (4.5)	2 (7.1)	
EASI score	-	7.23 ± 2.82	18.15 ± 1.87	28.96 ± 4.88	<0.001
IGA scale	-	1.24 ± 0.44	3.23 ± 0.43	4.21 ± 0.42	<0.001
Pruritus NRS	-	2.68 ± 1.11	5.23 ± 1.11	6.68 ± 1.36	<0.001

Values are presented as number (%) or mean ± standard deviation. DLQI: Dermatology Life Quality Index, EASI: Eczema Area and Severity Index, IGA: Investigator’s Global Assessment, LDL: Low density lipoprotein, NRS: Numerical Rating Scale, NS: Nonspecific. * Statistical analysis was made between atopic dermatitis groups.

**Table 2:** Pearson’s correlation coefficients of clinical and laboratory values.

Value	EASI	IGA	Pruritus_NRS	BMI	Total IgE	EOS
EASI						
Coefficient of correlation	1	0.944 **	0.698 **	0.077	0.455 **	0.263 *
*p* value		0.000	0.000	0.512	0.000	0.023
N	75	75	75	75	75	75
IGA						
Coefficient of correlation	0.944 **	1	0.770 **	0.054	0.459 **	0.295 *
*p* value	0.000		0.000	0.644	0.000	0.010
N	75	75	75	75	75	75
Pruritus NRS						
Coefficient of correlation	0.698 **	0.770 **	1	−0.024	0.233 *	0.286 *
*p* value	0.000	0.000		0.840	0.044	0.013
N	75	75	75	75	75	75
BMI						
Coefficient of correlation	0.077	0.054	−0.024	1	0.048	−0.036
*p* value	0.512	0.644	0.840		0.684	0.757
N	75	75	75	75	75	75
Total IgE						
Coefficient of correlation	0.455 **	0.459 **	0.233 *	0.048	1	0.210
*p* value	0.000	0.000	0.044	0.684		0.070
N	75	75	75	75	75	75
EOS						
Coefficient of correlation	0.263 *	0.295 *	0.286 *	−0.036	0.210	1
*p* value	0.023	0.010	0.013	0.757	0.070	
N	75	75	75	75	75	75

BMI: body mass index, EASI: Eczema Area and Severity Index, EOS: eosinophil count, IGA: Investigator’s Global Assessment, N: number of subjects, NRS: Numerical Rating Scale. ** *p* < 0.01, * *p* < 0.05.

**Table 3:** Clinical characteristics of severe atopic dermatitis patients undergoing dupilumab treatment.

Value	Baseline	Week 16	Week 52	*p* Value *
EASI score	28.96 ± 4.88	4.01 ± 1.83	2.63 ±1.72	<0.001
IGA scale	4.21 ± 0.42	1.43 ± 0.50	1.07 ± 0.60	<0.001
Pruritus NRS	6.68 ± 1.36	3.45 ± 1.02	2.01 ± 1.12	<0.001
Body mass index	22.19 ± 2.13	22.43 ± 2.12	21.58 ± 2.11	NS
Adiponectin (ng/mL)	3524 ± 1370	3382 ± 1355	3778 ± 1600	NS
Total IgE (IU/mL)	1140.1 ± 950.6	1084 ± 937.6	1010 ± 895.8	<0.05
Blood eosinophils count (%)	7.40 ± 2.21	7.29 ± 2.18	7.35 ± 2.54	NS
Total cholesterol (mg/dL)	183.74 ± 39.88	188.45 ± 41.69	179.22 ± 36.41	NS
LDL-cholesterol (mg/dL)	133.24 ± 30.26	137.15 ± 30.85	127.49 ± 29.22	NS

Values are presented as number (%) or mean ± standard deviation. EASI: Eczema Area and Severity Index, IGA: Investigator’s Global Assessment, LDL: Low density lipoprotein, NRS: Numerical Rating Scale, NS: Nonspecific. * Statistical analysis was carried out using repeated measure ANOVA.

## Data Availability

The datasets generated during and/or analyzed during the current study are available from the corresponding author on reasonable request.
